# Enabling dynamic network analysis through visualization in TVNViewer

**DOI:** 10.1186/1471-2105-13-204

**Published:** 2012-08-16

**Authors:** Ross E Curtis, Jing Xiang, Ankur Parikh, Peter Kinnaird, Eric P Xing

**Affiliations:** 1 Joint Carnegie Mellon, University of Pittsburgh PhD Program in Computational Biology, Pittsburgh, PA, USA; 2 Lane Center for Computational Biology, Carnegie Mellon University, Pittsburgh, PA, USA; 3 Machine Learning Department, Carnegie Mellon University, Pittsburgh, PA, USA; 4 Human Computer Interaction Institute, Carnegie Mellon University, Pittsburgh, PA, USA

**Keywords:** Visualization, Dynamic network analysis, Gene expression analysis

## Abstract

**Background:**

Many biological processes are context-dependent or temporally specific. As a result, relationships between molecular constituents evolve across time and environments. While cutting-edge machine learning techniques can recover these networks, exploring and interpreting the rewiring behavior is challenging. Information visualization shines in this type of exploratory analysis, motivating the development ofTVNViewer (http://sailing.cs.cmu.edu/tvnviewer), a visualization tool for dynamic network analysis.

**Results:**

In this paper, we demonstrate visualization techniques for dynamic network analysis by using TVNViewer to analyze yeast cell cycle and breast cancer progression datasets.

**Conclusions:**

TVNViewer is a powerful new visualization tool for the analysis of biological networks that change across time or space.

## Background

The rapid development of high-throughput technology and increasing amounts of biological data promises greater insight into the complex interactions that govern cellular function. In particular, gene expression measurements can be used to infer network relationships between genes in a cell, potentially uncovering important interactions that perturb the cellular state [1-4]. Understanding these network relationships between genes can lead to greater insight into cellular processes, such as the cell cycle or disease progressions [5]. Traditionally, gene networks have been analyzed as static entities. However, biological processes such as development and disease progression evolve over time and react to changing environments. Representing these dynamic interactions with a single static network limits the biological insights that can be derived from analysis. Recently, biologists have attained a deeper knowledge of the functional and regulatory underpinnings of complex biological processes by studying dynamic gene-gene relationships [6-9]. In addition, recent algorithmic advancements allow these time-varying networks to be reverse engineered from a time series of molecular profiles. As techniques in dynamic network analysis continue to advance, tools that can visualize these complex networks will become increasingly important to understanding the systematic rewiring of the transcriptional regulatory circuitry that controls cell behavior.

**Figure 1:**
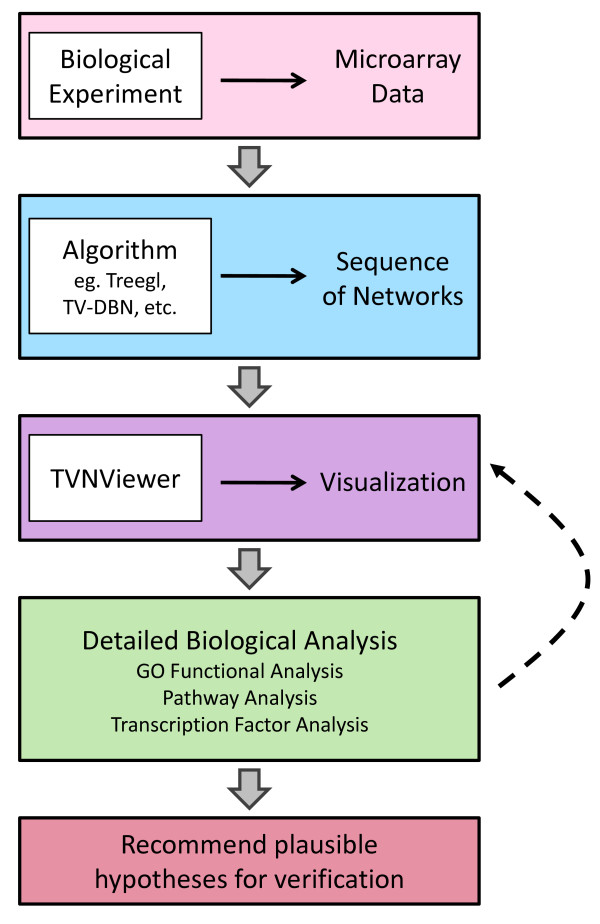
**Dynamic network analysis pipeline.** A dynamic network analysis consists of several stages beginning with data collection and leading to generating new hypotheses for further study. First, gene expression data (usually microarray data) is collected across several time points or tissue types. Once the data is preprocessed, machine learning techniques are employed to determine relationships between genes. As a result, a series of networks are created that can then be explored using TVNViewer. Detailed biological analyses are then carried out leading to specific hypotheses that can then be validated experimentally.

Dynamic network analysis begins with data collection and the creation of a series of gene-gene interactions (networks) from the data (Figure 1). Dynamic gene expression data is generally available as microarray samples that are collected over a time course or under multiple conditions. Many cutting-edge machine learning techniques are available to fully leverage the information stored within the data to create a series of related, evolving gene networks. Here, we list a few of these strategies. TESLA and KELLER builds off sparse regression techniques [10,11] and TV-DBN [12] estimates a chain of evolving networks using time-varying dynamic Bayesian networks. In addition, Robinson and Hartemink suggested learning a non-stationary dynamic Bayesian network using Markov Chain Monte Carlo sampling [13] and Lozano *et al.*proposed a different approach that uses the notion of Granger causality to model causal relationships among variables over time [14]. In contrast to linear time-varying networks, Treegl is a method for analyzing networks that evolve over tree-shaped genealogies (such as stem cell differentiation) [15]. Each of these strategies can be used to recover a series of networks from dynamic gene expression data for further analysis. 

Once a series of networks is available for analysis, these networks must be explored to find the subtle (and obvious) changes in network topology. Analysts can examine the changing network topology to find key regulators that drive the network evolution. At this point, the focus becomes more exploratory than query driven. Information visualization, “the use of computer-supported, interactive visual representations of data to amplify cognition,” as a field, touts its strengths at generating exploration-based insights, explanatory and persuasive interaction, and aesthetic representations [16]. Visualization techniques excel at providing an explanation of the overall structure of the data or finding weak or unexpected patterns most easily recognized by humans [17].

Because visualization naturally enables gene network analysis, many visualization tools have been developed to explore biological networks including Cytoscape [18], Osprey [19], VisANT [20], and Graphle [21]. Although many tools exist, the state-of-the-art tools in biological network analysis do not support the exploration of dynamic networks [22,23]. While information visualization researchers have developed and evaluated techniques for dynamic network analyses of numerous kinds in other contexts, including social networks [24,25], internet traffic networks [26], and even literature networks [27], these tools are not easily applicable in the biological context . Additionally, in our own experience exploring dynamic gene networks, we have found that multiple networks need to be visualized simultaneously and in real-time. To explore these networks, analysts must conveniently load and view a large number of networks and rapidly switch between networks to compare the topologies. Thus, given our experience and current visualization research, we have found that the visualization tools available for gene network analysis, such as Cytoscape, are insufficient to support the analysis of a large number of rewiring networks.

We present TVNViewer, an online visualization toolspecifically designed to support the discovery of spatial or temporal changes in network topology via exploration [28]. In addition to facilitating exploratory analysis, TVNViewer allows analysts to create the intuitive visualizations required to present their discoveries. In this paper, we demonstrate how the visualizations in TVNViewer facilitate dynamic network analysis through the analysis of two real datasets. The first dataset is a yeast (*Saccharomyces cerevisiae*) microarray dataset that contains 5610 genes measured at 25 time points across two cell cycles [29]. The networks at each time point have been recovered using Time-Varying Dynamic Bayesian Networks (TV-DBN) [12]. The second dataset is a breast cancer progression and reversal dataset [30]; breast cells grown in a 3D culture begin as normal cells, become malignant (cancerous), and are then reverted by drugs that inhibit various signaling pathways. The networks have been recovered using Treegl [15]. TVNViewer can be used to expose the similarities and differences of these cells states to reveal the effectiveness of various drugs.

The outline of the paper is as follows: we first discuss the implementation and design of TVNViewer and then show how the visualizations available in TVNViewer enable the analysis of the yeast and breast cancer datasets through several visualization strategies. Finally, we demonstrate the power of dynamic network analysis in TVNViewer via biological analysis of the breast cancer and yeast datasets.

## Implementation

TVNViewer runs as a freely available online visualization tool, accessed from http://sailing.cs.cmu.edu/tvnviewer. We present several resources for analysts to learn how to use TVNViewer: extensive online documentation, video tutorials, and five example preloaded networks. Analysts who create an optional login can store up to ten datasets directly on the TVNViewer website. However, all TVNViewer functionality is available without a login through a temporary session. Data for TVNViewer is stored securely on the website in a MySQL database. Analysts can upload data onto the website as described in the online documentation.

TVNViewer itself is implemented using Adobe ActionScript, and thus runs on all major browsers with the freely-available Adobe Flash plug-in. TVNViewer is an open-source project; the source code for TVNViewer can be downloaded from the main website. To implement TVNViewer, we built off of Flare, an easily-customized, open-source web-visualization project (flare.prefuse.org).

In addition to providing different visual representations of the data, TVNViewer allows the analyst to customize network views to the analyst’s preferred visual representation. Specifically, the analyst can adjust the size of the data nodes, choose to have the data nodes sized based on degree, adjust the font size of the labels, or change the visual thickness of the edges. Based on the size of the analyst’s screen, TVNViewer dynamically resizes the visualization to ensure that all labels and nodes fit within the visualization window. The opacity of each edge in TVNViewer represents its weight in the network and can be adjusted by the analyst. Additionally, the analyst can select what edges and node labels are visible in the visualization. For example, consider the case where a network has many edges with a low weight. In this case, the analyst increases the minimum edge threshold and all edges below this threshold disappear, revealing the strongest interactions. Another scenario is where the analyst is interested in only a handful of genes or gene groups. In this scenario, the analyst can remove all other labels from the visualization, highlighting the specific genes of interest. Providing customizable, interactive visualizations like these allows analysts to enhance their own cognition by putting their knowledge into the analysis. Rather than constantly having to remember numeric or ordinal values for edge weights, for example, the visualization off-loads those considerations to the visual cortex, allowing the analyst to focus on analytic activities rather than the trivia of edge weights which are only valuable for the analyst in so far as they generate insights [31].

## Results and discussion

In this section, we highlight some ofTVNViewer’s visualizations available for dynamic network analysis. In each case, we use the yeast or breast cancer data to show how an analyst would use TVNViewer to discover patterns and information in the recovered set of networks. After the demonstrations, we will discuss the results of using TVNViewer for dynamic network analysis.

### One-level network circle view

An important challenge in dynamic network analysis is the recognition of subtle changes in the network topology over time. In the one-level network circle view, the analyst sees all the genes in the dataset aligned in a circle layout. The genes are represented by circles (nodes) and the connections between genes are represented by edges (lines between nodes). The genes areclustered to minimize the number of edges going across the circle, keeping most edges local to tight clusters of genes around the edge of the circle. Genes are colored by this clustering; details are provided in the online documentation describing how this is done. Also, the analyst can use the tree view to view the sorting tree of how the nodes were clustered.

**Figure 2:**
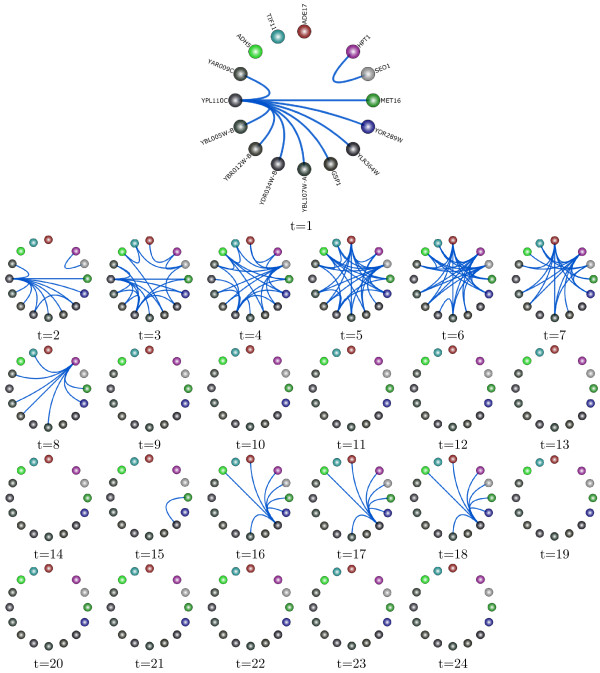
**One-level gene network view.** We use the one-level gene network view in TVNViewer to explore the rewiring of a subnetwork of genes generated from the yeast cell cycle data. The network rewires across two different cell cycles. The first cycle occurs during t = 1-12, and the second cycle is from t = 13-24. We can readily observe that the network is most active during the initial phases of the cell cycle, which coincide with the G1 phase. While there is overlap between the timing of phases, G1 occurs at the beginning of the cell cycle, so roughly time points 1–6 and 13–18.

In the one-level network circle view, the analyst can step through the sequence of networks in real time to explore the rewiring of the gene networks. We demonstrate this feature in Figure 2, where we show a subnetwork of genes at 24 time points from a large network derived from yeast gene expression data. The top graph in Figure 2 represents the gene network at Time 1, and all nodes are labeled by the names of the genes they represent. To enhance the figure’s readability, we have utilized TVNViewer’s option to remove gene name labels in the graphs representing the other time points. The 24 time points in this figure represent two cell cycles where the first occurs between time point 1 and 12 and the second occurs between time point 13 and 24. The one-level gene network view in TVNViewer makes the appearance and disappearance of edges in the network readily accessible to the analyst, without the awkward integration or customization required by other network visualization tools. The analyst can quickly identify that this particular network is active in the beginning of each cell cycle which corresponds to the G1 phase of the cell cycle.

### Two-level network with GO annotations

**Figure 3:**
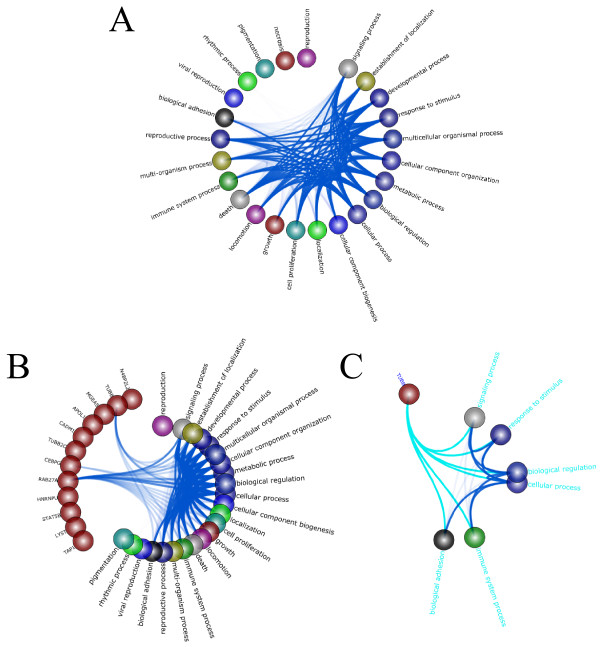
**Two-level network view.** In TVNViewer’s two-level network view, the genes are grouped by GO category, and the analyst can explore the overall topology of the network or zoom into the small-scale gene-gene interactions. **A**) An overview of the network. Genes involved in cell death and proliferation are especially active. **B**) Groups of interest can be expanded to reveal the genes involved in the group and their interactions with other groups. To illustrate this feature, we have expanded the “necrosis” group. **C**) By selecting genes, we can observe the interactions of specific genes (in this case *TUBB*) with the rest of the network.

Often, there are more genes in the network than can be visualized by using circle view. In this case, it is more helpful to group similar genes by function (*i.e.* gene ontology (GO) groups) and then visualize the interactions amongst the groups. TVNViewer provides a two-level network view specifically designed to allow high level exploration of the network at the group level, while still being able to zoom in to explore individual gene interactions. Consider analyzing a T4 malignant breast cancer cell network with 5440 genes (nodes), generated using Treegl [15]. A two-level network view using second level GO biological process groups is shown in Figure 3A. One can zoom in on a specific group, such as “necrosis” (Figure 3B), revealing the genes associated with that group. The analyst can zoom even further by selecting a particular gene to reveal its specific interactions. For example Figure 3C shows that the *TUBB* gene (tubulin beta) interacts with genes from many groups, most notably the signaling process and biological adhesion groups. This makes sense since *TUBB* encodes proteins that are important to GTP binding and GTPase activityin addition to its involvement in the structure of the cytoskeleton. Thus, the two-level view provides the analyst with both a high level perspective of the networks while simultaneously allowing him to focus on particular genes. 

### Directed graphs

**Figure 4:**
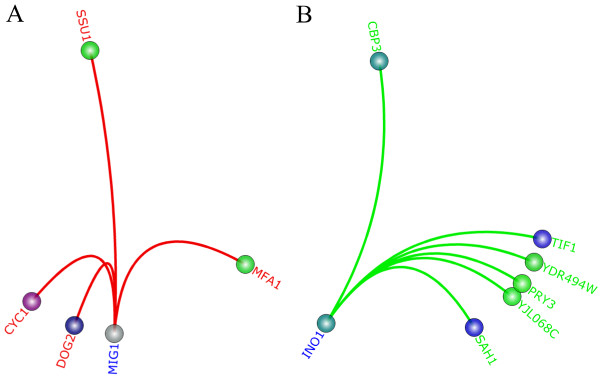
**Directed edges.** TVNViewer also supports datasets with directed or undirected edges. In this case, we show two genes in the yeast dataset with different edge patterns. **A**) *MIG1*is shown with only in-edges colored red, suggesting that it is regulated by multiple genes. **B**) *INO1* is shown with only out-edges, suggesting that it regulates the expression of the genes highlighted in green.

TVNViewer can be used to visualize both directed and undirected graphs. Directed graphs are valuable if an analyst is interested in cases where the direction of the edge is significant, such as in a regulatory cascade. The initial layout of the graph is not changed in the case of directed graphs for the circle and force views. However, as the analyst hovers over different genes, TVNViewer will highlight all of the gene’s in-edges in red, out-edges in green, and bidirectional edges in cyan. If an analyst is interested in one particular gene or gene group, he can select that particular node and TVNViewer will isolate that node and show only the genes connected to it. For example, in Figure 4A, we have selected *MIG1*in the yeast dataset; all the edges connected to it are highlighted in red indicating that they are in-edges, implying that they may have a regulatory relationship with *MIG1*. However, in Figure 4B, the selected node *INO4* has only out-degree nodes since the edges connected to it are green. This suggests that these genes may be regulated by *INO4*. These regulatory relationships may change across time or space, and the analyst can use TVNViewer to trace these relationships using directional information.

### Stack view

**Figure 5:**
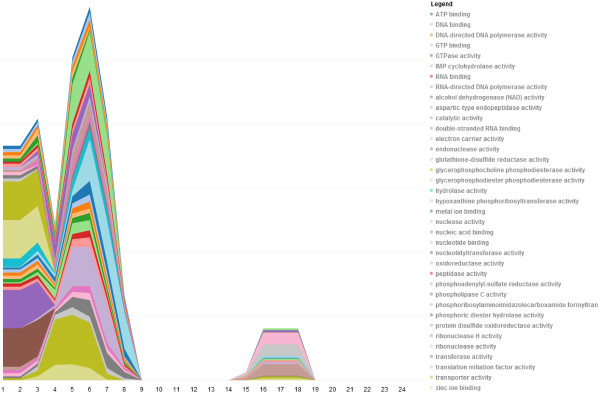
**Stack view.** The stack view allows analysts to get a general overview of how the gene degree of the entire network changes across time. In the yeast cell cycle data, we observe two distinct time periods with high activity. We can also see that different functional groups contribute to the height of the stack differently at different times. By hovering over these groups, the analyst can identify the functions and observe their evolution. While there is overlap between the timing of phases, G1 occurs at the beginning of the cell cycle, so roughly time points 1–6 and 13–18. You can see that these groups are mostly active in G1-phase.

While the circle layouts allow analysts to understand how gene networks rewire over time or space, the stack view visualization is better fit for exploring how specific interactions between genes or gene ontology groups change over time. For instance, we would like to be able to view how the biological functions of the network change over time, such as over the course of a cell cycle. This can be done by grouping the genes by their GO functional group to visualize with the stack view (Figure 5). In this view, the out-degree of each GO category is stacked, one on top of the other. Thus, the variation in individual GO categories is clear, and the overall variation in out-degree is emphasized. This visualization clearly shows that the overall network is active during G1 phase and we observe that genes in the GO categories: ATP binding, electron transport chains, and phospholipase C activity are especially active. This is expected as these are all functions involved in cellular respiration, which is the signature activity of the G1 phase of the cell cycle. By hovering over the GO category in the stack; both the GO category and its degree at the given time point is displayed.

**Figure 6:**
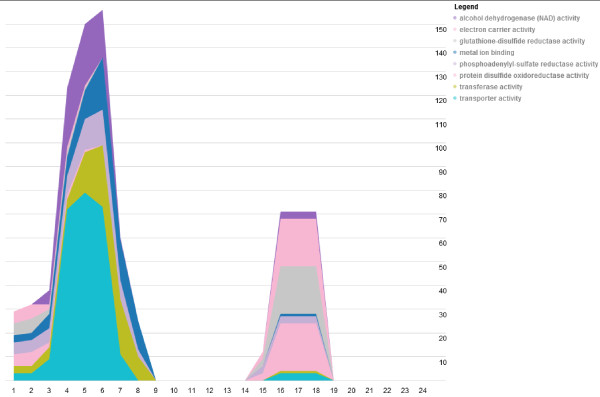
**Using filters in the stack view.** TVNViewer allows analysts to filter the stack view to isolate specific functional groups or specific genes and how they evolve across time. Here, the analyst considers how the degree of genes involved in electron carrier activity, alcohol dehydrogenase, and other enzymatic activities change across the two yeast cell cycles.

**Figure 7:**
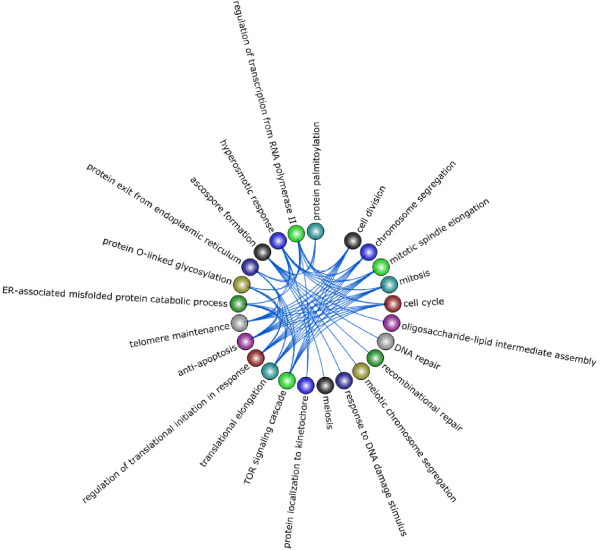
**G2M active genes in yeast.** The subnetwork shown is a selection of yeast genes that were found to be active during the G2M phase. As a result, the functional groups describe biological processes that occur in the G2-phase of the cell cycle and the final phase which is mitosis. Specifically, groups like DNA repair are indicative of G2-checkpoint and groups such as chromosome segregation annotate genes involved in mitosis.

The analyst can select specific GO categories of interest by listing them using the filter box, or by simply selecting them on the plot. Additionally, if the analyst is interested in specific genes, he can go past the group level and generate stack plots of genes of interest. Although it is relatively simple to implement selection and filtering functions in a visualization, the impact provided by these features is substantial. By allowing analysts to rapidly and simply subset their data while highlighting items of interest, we allow analysts to play “what if” scenarios, which may combine a number of highlights or filters. These visualization features, comparable to dynamic queries, drastically lower the cost of exploring and experimenting with the data and evaluating the outcome of varying queries in comparison to database queries or other approaches [32]. In Figure 6, we use filters on the stack view to show recurring GO groups including electron carrier activity, alcohol dehydrogenase (NADH) activity, and various enzymatic processes. Figure 7 shows that these groups are active between time points 1–8 and 14–19. The timing is consistent with G1-phase which occurs at the beginning of each cell cycle. This observation is expected biologically; we expect that the cell is growing during G1, and thus cellular respiration, which requires electron carrier activity, and NADH activity, and other enzymatic activities are occurring. 

### Analysis of temporally dependent gene-gene interactions across the yeastcell cycle

Budding yeast (*Saccharomyces cerevisiae)* serves as an excellent model for dynamic network learning because the molecular mechanisms of the cell cycle control system are well known [33]. Budding yeast follows the eukaryotic cell cycle, which is divided into 4 distinct phases [34]. The first is G1-phase (gap 1), which is the interval between mitosis and DNA synthesis where the cell is actively growing. This is followed by S-phase (synthesis) during which DNA replication occurs. The cell continues to grow during G2 (gap 2) and then divides in the M or mitosis phase. For the purpose of this study, we group the G2 and M phase and refer to it as G2M.

Studying the yeast cell cycle is a fitting scenario for utilizing TVNViewer as both an exploratory tool and a method of validation.We first generate a series of networks across time from yeast gene expression data using TV-DBN [12]. Then we select subnetworks that are active during certain cell cycle phases and observe their temporal activity as it relates to their function. For example, Figure 7 shows a network with genes that were found to be active during the G2M-phase. Here, we observe functional groups that are clearly relevant to M-phase such as chromosome segregation, mitotic spindle elongation, and telomere maintenance. In addition, we observe GO groups like DNA repair, recombinational repair, and response to DNA damage stimulus which are indicative of G2-phase. One of the major checkpoints occurs in G2 phase, whereby cells are arrested in response to damaged or unreplicated DNA [34]. Thus, we can conclude that these functions are aligned with what we expect from genes that are active in G2M.

**Figure 8:**
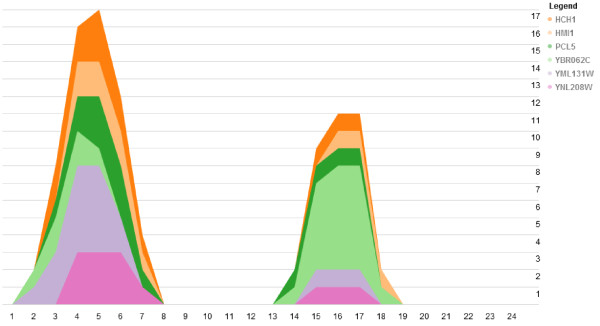
**Genes active during S phase in yeast.** The plot shown is generated from a selection of yeast genes active in S-phase. The stack view shown illustrates the recurring activity of particular genes over time. Here, we can easily identify the time and shape of interaction patterns that repeat across cell cycles. The peak times are around time points 4–5 in the first cell cycle and 16–17 in the second cell cycle.

**Figure 9:**
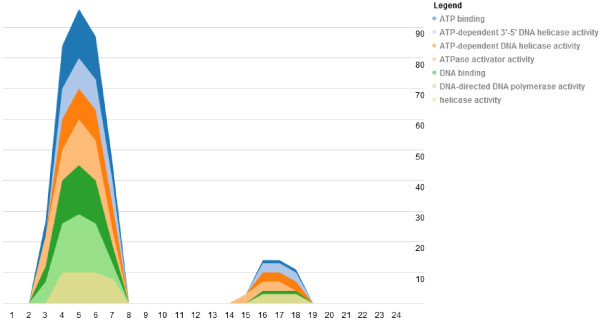
**Gene functional groups active during S phase in yeast.** By annotating the genes from Figure 10 using GO functional groups, we can observe the recurring functional groups. In this example, DNA binding, helicase activity, and DNA-directed DNA polymerase activity are all molecular mechanisms that we expect to occur during S-phase.

**Figure 10:**
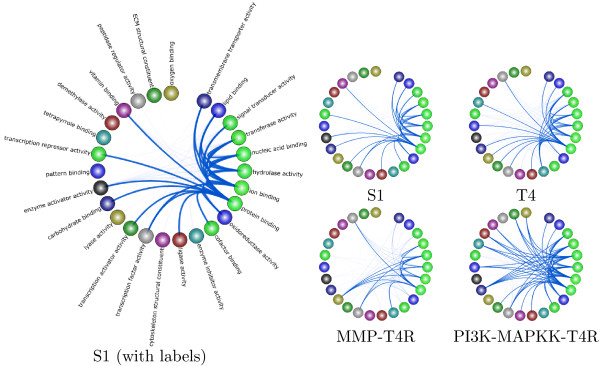
**Breast cancer analysis using GO molecular function annotation.** Here we present a summary of our results from the analysis of the breast cancer data using the GO molecular function annotations for the genes. The network derived from the original cells is denoted by S1, the network from the cancer cells is denoted T4, and the networks from the reverted cells are labeled MMP-T4R and MAPKK-T4R.

An important characteristic of cell cycle data is that it is repetitive. Thus, we should observe recurring patterns in the time-varying networks. Figure 8 shows a set of genes, active in S-phase. The colored layers of plots clearly indicate that the interactions between the genes repeat over the two cell cycles; the first cell cycle occurs between time points 1–12 and the second during time points 13–24. If we take the same subnetwork shown in Figure 8 and annotate the genes using GO functional groups, we can observe which groups are active over the time series (Figure 9). Similar to Figure 10, the colored layers show the GO groups repeat across the two cell cycles. The GO terms listed are also relevant to S-phase as they indicate the presence of genes involved in DNA binding, helicase activity and ATP binding.

From this preliminary overview of the functional significance of the genes provided by TVNViewer, we can then focus on particular genes and investigate supporting biological literature that can both confirm and explain why these genes interact. For instance, the gene *HMI1* was found to be a DNA helicase and experimental results indicated that it localized in the mitochondria and was required for the maintenance of the functional mitochondrial genome [35]. The unwinding activity of the helicase requires ATP hydrolysis and has a 3′ to 5′ polarity [36]. Another gene in the subnetwork is *YNL208W*. While not much is known about the function of*YNL208W*, the protein was detected in purified mitochondria [37]. Interestingly, experimental evidence places both *HMI1* and *YNL208W* at the same cellular location, supporting the prediction by our network that these genes interact.

Studying developmental processes such as the yeast cell cycle requires the integration of temporal and functional information. By using TVNViewer, we identify the recurring patterns of the gene subnetworks in S-phase.We also find that the functional roles of the genes in the network are consistent with the timing of network activity. This analysis canguide the exploration of biological literature to link the gene-gene interactions and formulate a summarizing regulatory mechanism.

### Exploring the progression and reversal of breast cancer

Using TVNViewer, we also investigate the progression and reversion of breast cancer cells using dynamic network analysis. Functional analysis of 3D culture models of breast cancer has led to a deeper understanding of the effect of a cell’s microenvironment on tumorgenesis and metastasis [30]. It was found that micro-environmental factors and signaling pathways have a dramatic influence on the growth dynamics and malignancy of the cells [38,39]. Furthermore, treatment with inhibitors of various signaling molecules causes reversion of T4 cells into morphologically-normal-looking cells (T4R cells). Our objective is to analyze the functional differences amongst the different cell states.

**Figure 11:**
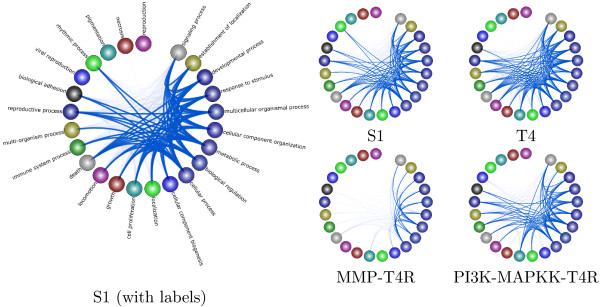
**Breast cancer analysis using GO biological process functional annotation.** Here we present a summary of our results from the analysis of the breast cancer data using the GO biological process functional annotations for the genes. We present the network derived from the original cells (S1), a network derived from the cancer cells (T4), and then networks derived from the reverted cells. Nodes signify GO biological process groups and the relative thickness of the edges between groups represents the number of genes that interact between the two groups.

**Figure 12:**
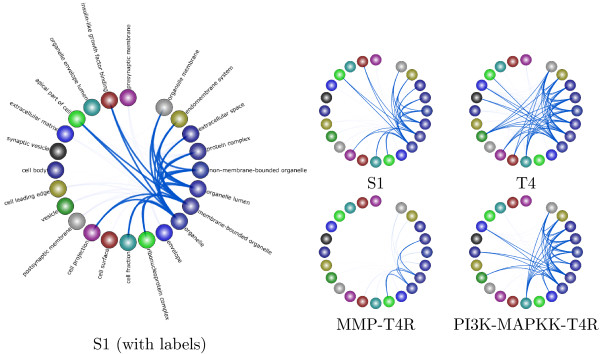
**Breast cancer analysis using GO cellular component annotation.** Here we present a summary of our results from the analysis of the breast cancer data using the GO cellular component annotations for the genes. The network derived from the original cells is denoted by S1, the network from the cancer cells is denoted T4, and the networks from the reverted cells are labeled MMP-T4R and MAPKK-T4R.

We first used Treegl [15] to reverse engineer gene networks for each cell state. As shown in Figure 11, compared to S1 cells, T4 cells display increased activities in cell proliferation and locomotion, both of which are indicative of cancer. Furthermore, we see that that the T4 network exhibits significantly more interaction with the extracellular matrix and other components related to the cell membrane such as the vesicle (Figure 12). This is expected since it has been found that a cell’s interaction with its microenvironment affects tumorgenicity and metastasis [40]. Finally, one can see that the T4 network also displays increased signal transducer activity (Figure 10). Signal transducers and activators of transcription, especially those associated with cytokine and growth factor activity have been implicated in tumorigenesis [41]. 

As we can readily observe from the figures, the T4R cells are different from the S1 and T4 cells, but are also distinct from each other. The MMP-T4R network is very sparse and thus has few interactions. Notably,cell proliferation and other indicators of cancer are absent in MMP-T4R cells. On the other hand, the PI3K-MAPKK-T4R cells still display considerable cell proliferation and interaction with the extracellular matrix. PI3K-MAPKK –T4R cells also exhibit more activity such as tetrapyrole binding, demethylase activity and carbohydrate binding, all of which are absent in the other cell states. Collectively, these data suggest that although T4 cells can be morphologically reverted back to the normal-looking T4R cells, the underlying molecular mechanisms in the reverted cells are different from those in either S1 or T4 cells and from one another.

## Conclusions

The cellular mechanisms responsible for progression through the cell cycle or the development of disease are complex and dynamic. Thus, many machine learning approaches have been designed to construct dynamic networks that model these processes. To fully exploit the information captured in these networks, we must have the visualization framework to simultaneously analyze a series of networks. However, current network visualization tools require extensive adaptation to explore a time series of networks. In many cases, this would require the generation of separate network visualizations, which cannot be easily compared and explored in real time.

In this work, we have demonstrated TVNViewer, a new visualization tool built for exploring the dynamic relationships between genes across a time series or in response to environment or disease. TVNViewer provides a clean interface that can be used to enable high-level functional and topological analysis in addition to highlighting more subtle network interactions over time. It facilitates a convenient and intuitive analysis of a yeast and breast cancer dataset that would not be possible using other gene network visualization tools. To conclude, TVNViewer can enable researchers to leverage the networks produced by machine learning and statistics methods through presenting the temporal context and combination of gene-level and functional-level information to allow for extensive biological analysis and interpretation.

## Availability and requirements

·**Project name:** TVNViewer

·**Project home page:**http://sailing.cs.cmu.edu/tvnviewer

·**Operating system(s):** Platform independent

·**Programming language:** ActionScript

·**Other requirements:** Adobe Flash Player

·**License:** Noncommercial research use

·**Any restrictions to use by non-academics:** license needed

## Competing interests

The authors declare that they have no competing interests.

## Authors’ contributions

REC developed the software and drafted the manuscript. JX and AP tested the software, performed the biological analysis, and drafted the manuscript. PK provided a visualization perspective and drafted the manuscript. EPX directed the project and drafted the manuscript. All authors read and approved the final manuscript.
